# Supplementary material for: Reduced Glx and GABA Inductions in the Anterior Cingulate Cortex and Caudate Nucleus Are Related to Impaired Control of Attention in Attention-Deficit/Hyperactivity Disorder
Source: Int J Mol Sci. 2022 Apr 23;23(9):4677. doi: 10.3390/ijms23094677 (PMC9100027; doi:10.3390/ijms23094677)
Supplement: Supplementary file 1 [file ijms-23-04677-s001.zip › ijms-1650823-supplementary.pdf]

Supplementary information

Supplementary Table S1. Demographic and clinical characteristics of study participants.

**Categories**	**Mean (SEM)**
**Age**	
CONTROL	41.0 (2.36)
ADHD	42.6 (1.73)
**Categories**	**Number of observations**
**Gender**	
CONTROL	4/10 (M/F)
ADHD	4/14 (M/F)
**Highest degree obtained**	
CONTROL	High school: 2<br>College or higher: 14
ADHD	High school: 1<br>College or higher: 17
**Language background**	
CONTROL	Monolingual: 12<br>Non-monolingual:4
ADHD	Monolingual: 15<br>Non-monolingual: 3
**Employment**	
CONTROL	Full-time: 9<br>Part-time or self-employed: 4<br>Unemployed: 3
ADHD	Full-time: 11<br>Part-time or self-employed: 4<br>Unemployed: 3
**Medications**	
CONTROL	N/A
ADHD	Methylphenidate: 1<br>Dextroamphetamine: 2<br>Ritalin: 1

Supplementary Table S2. GABA and Glx concentrations shown in institution units. Concentrations are shown as mean with standard deviation in the parentheses. Block1: non- task block; Block2: auditory task; Block3: Stroop task; Block4: Flanker task.

	**CONTROL**			**ADHD**		
	Concentrations	Confidence interval		Concentrations	Confidence interval	
		Upper	Lower		Upper	Lower
**GABA**						
Block1	2.50 (0.29)	2.66	2.35	2.50 (0.32)	2.66	2.35
Block2	2.55 (0.29)	2.71	2.40	2.63 (0.28)	2.77	2.50
Block3	2.59 (0.23)	2.71	2.47	2.55 (0.35)	2.77	2.38
Block4	2.62 (0.30)	2.78	2.46	2.58 (0.32)	2.74	2.43
**Glx**						
Block1	5.84 (0.95)	6.34	5.33	5.69 (0.70)	6.03	5.35
Block2	6.60 (0.96)	7.11	6.08	6.15 (0.73)	6.51	5.80
Block3	6.73 (0.63)	7.06	6.39	6.35 (0.74)	6.72	5.98
Block4	6.90 (1.02)	7.44	6.36	6.33 (0.96)	6.81	5.85

Supplementary Table S3. The error rates in the Auditory and Stroop task.

	**Auditory**	**Stroop**
**CONTROL**	53.8±3.33	11.23±2.02
**ADHD**	42.9±2.92	15.15±2.44
**Statistical results**	$H_{(1)}$=0.57, p=0.448	$H_{(1)}$=1.220, p=0.269

Data are shown as mean with standard deviation. Kruskal-Wallis test was used to assess the differences in error rates between CONTROL and ADHD groups.

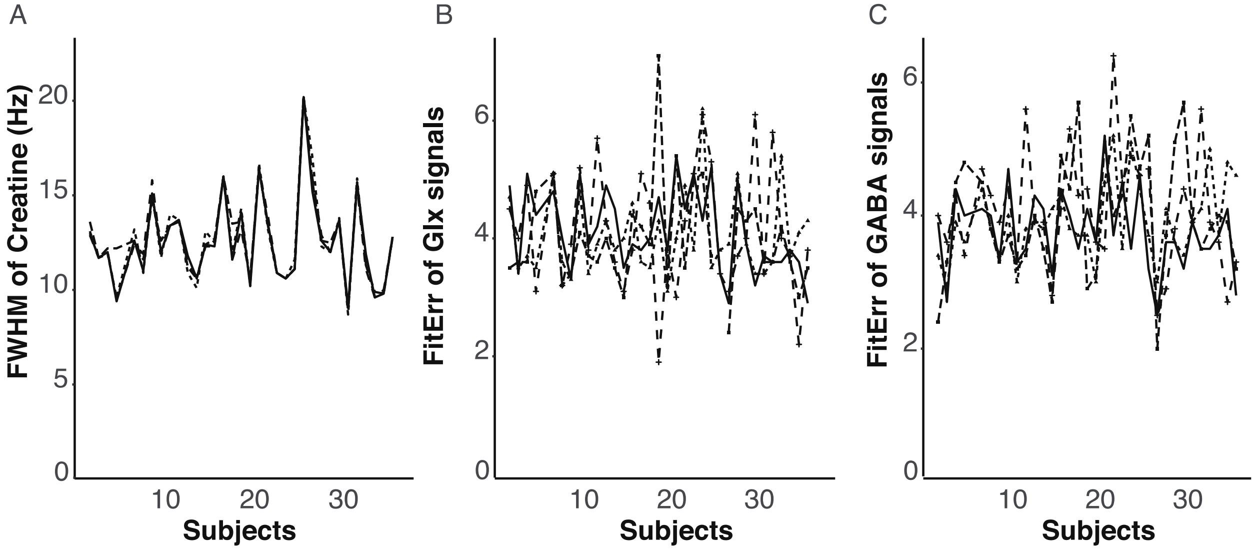

Supplementary Figure S1. (A) FWHM of creatine signals, (B) fiterror of Glx signals, and (C) fiterror of GABA signals.

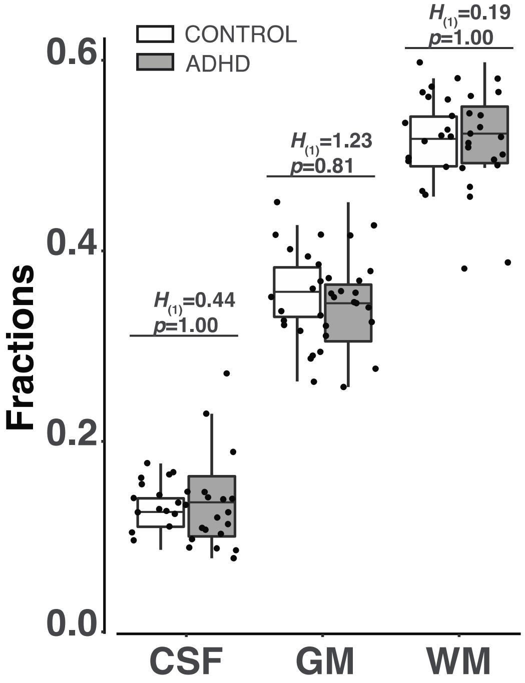

Supplementary Figure S2. Fractions of gray matter (GM), white matter (WM), and cerebrospinal fluid (CSF) within the MRS brain voxel. Open bars represent subjects without ADHD and grey bars represent subjects with ADHD. Dots represent the data points from individual subjects in all figures. The upper

boundary of an individual box represents the 75th percentile and the lower boundary represents the 25th percentile of the value for an individual block. The horizontal line within the box represents the median in a respective block.
